# Acute phlegmonous esophagitis as a rare but threatening complication of chemoradiotherapy: report of a case

**DOI:** 10.1007/s00595-013-0536-2

**Published:** 2013-03-07

**Authors:** Hiroyuki Karimata, Tadashi Nishimaki, Akehiro Oshita, Masayoshi Nagahama, Hideaki Shimoji, Morihiko Inamine, Tadatsugu Kinjyo

**Affiliations:** 1 Department of Digestive and General Surgery, Graduate School of Medicine, University of the Ryukyus, 207 Uehara, Nishihara, Okinawa 903-0215 Japan; 2 Department of Gynecology, Graduate School of Medicine, University of the Ryukyus, Nishihara, Okinawa Japan

**Keywords:** Acute phlegmonous esophagitis, Chemoradiotherapy, Adverse event

## Abstract

Phlegmonous infection involving the digestive tract has been reported to have a poor prognosis. However, the pathogenesis and clinical features of acute phlegmonous esophagitis have remained unclear due to the rarity of the disease. We herein report a case of acute phlegmonous esophagitis that showed a fulminant course during chemoradiotherapy for uterine cancer. The patient developed septic shock 10 h after postprandial nausea and vomiting, and a computed tomographic scan showed diffuse thickening of the esophageal wall. Severe leukopenia that was refractory to the administration of granulocyte colony-stimulating factor persisted during the first few days. The patient fortunately survived after intensive treatment. The acute phlegmonous esophagitis of the present case might have been evoked and worsened by chemoradiotherapy due to its emetic and myelosuppressive adverse effects, respectively. Although its incidence is extremely rare, acute phlegmonous esophagitis may occur as a life-threatening complication of chemoradiotherapy.

## Introduction

Phlegmonous infection of the digestive tract has been reported to be a rare disorder that is associated with a high mortality rate. The most common organ to be involved by phlegmonous infection has been reported to be the stomach, and more than 100 cases of gastric phlegmon have been reported so far. Kim et al. reviewed 36 cases of phlegmonous gastritis that were reported during the period from 1973 to 2003, and calculated an overall mortality rate of 42 % [1]. However, the esophagus is rarely affected by this disease. An extensive literature survey found only 12 reported cases of phlegmonous esophagitis [2–13]. Because of the paucity of reported cases, the pathogenesis, clinical features, adequate management, and outcome of acute phlegmonous esophagitis (APE) are unclear.

We recently experienced a fulminant case of APE that occurred during chemoradiotherapy for carcinoma of the uterine cervix. A similar case of APE has never been reported. We herein report in this case of APE, together with the possible mechanisms by which the given chemoradiotherapy might have played a crucial role in the development of this serious disease. We also review the pertinent literature and summarize the clinical characteristics of APE.

## Case report

A 47-year-old female was referred to the Department of Gynecology of Ryukyu University Hospital with a diagnosis of stage IIb carcinoma of the uterine cervix, as defined by the International Federation of Obstetricians and Gynecologists classification in November, 2010. A computed tomography (CT) scan of the chest upon admission showed no abnormal findings of the mediastinum. Before resection, she underwent two courses of induction chemotherapy that consisted of intravenous infusion of paclitaxel (175 mg/m^2^) and cisplatin (50 mg/m^2^) on day 1 with an interval of 3 weeks between courses. She experienced mild nausea after each course of chemotherapy. Although her white blood cell (WBC) count was 6500 cells/μL prior to the chemotherapy, she had leukopenia after the chemotherapy: her WBC count decreased to 2900 cells/μL and 3700 cells/μL after the first and the second course, respectively. After the chemotherapy, the tumor volume was reduced to 19 % of the initial size observed on magnetic resonance imaging. Three weeks after the chemotherapy, chemoradiotherapy, which consisted of an intravenous infusion of paclitaxel (50 mg/m^2^) and cisplatin (50 mg/m^2^) on day 1, and concurrent radiotherapy, which was delivered in daily fractions of 2 Gy (for a total of 40 Gy) to the pelvic area, was started in order to further shrink the tumor.

**Fig. 1:**
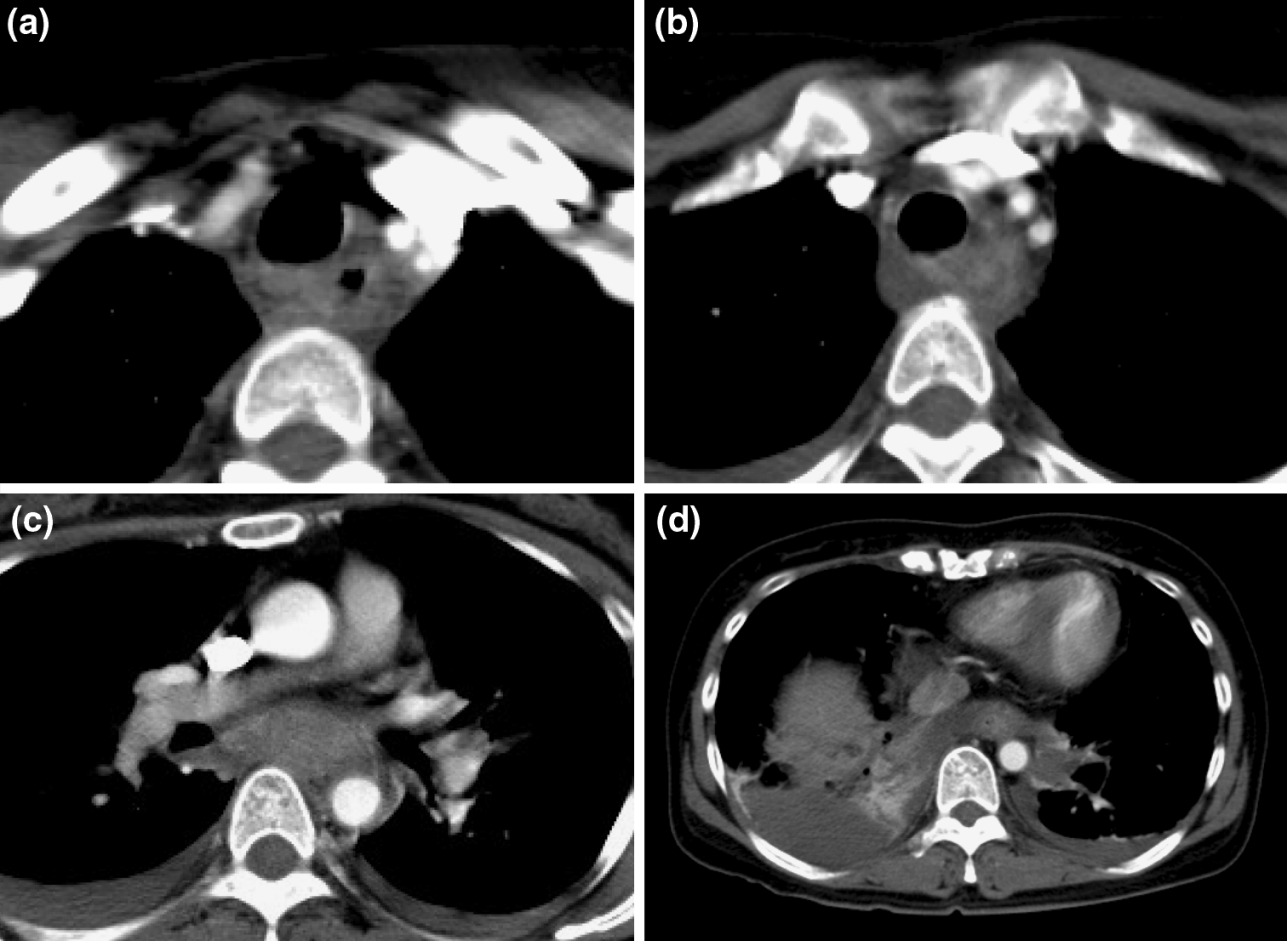
An enhanced computed tomography (CT) scan conducted on the day of onset showing diffuse thickening of the esophagus and edematous enlargement of the posterior mediastinum with bilateral pleural effusions. Sections are shown at the level of the cervical esophagus (**a**), the thoracic inlet (**b**), the mid-esophagus (**c**), and the lower esophagus (**d**). Note the low attenuation surrounded by a peripheral enhancing rim (**b**)

**Fig. 2:**
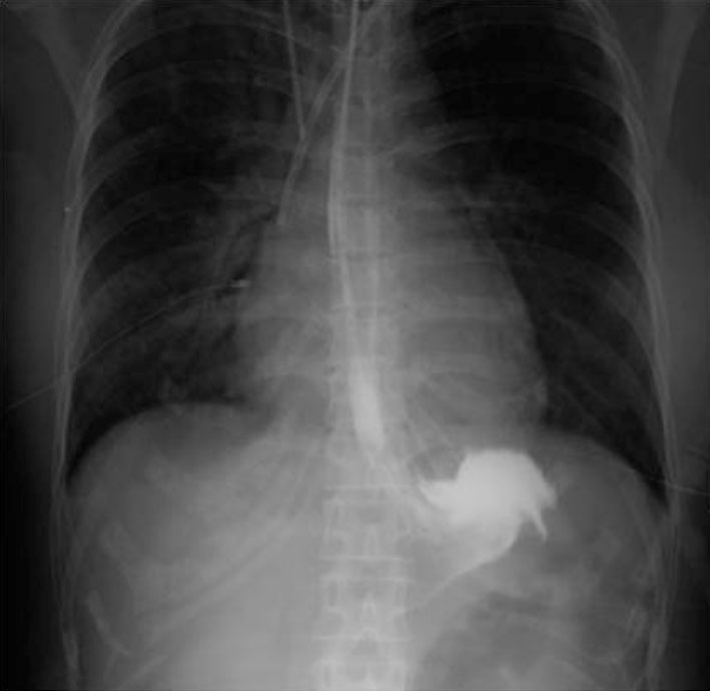
Esophagogastrography using water-soluble contrast medium injected through a nasogastric tube showed no evidence of esophageal perforation

Five days after the initiation of the chemoradiotherapy, the patient experienced nausea and vomiting soon after eating lunch. Three hours after lunch, she had severe and persistent epigastralgia. Ten hours later, dyspnea developed, which was soon followed by shock. At that time, a blood examination showed severe leukopenia (WBC: 600 cells/μL) and evidence of disseminated intravascular coagulation (DIC). She was suspected of having an acute pulmonary embolism, and therefore underwent a CT scan. The CT scan showed no evidence of a pulmonary embolism. However, a diffuse edematous enlargement of the posterior mediastinum with a thickened esophageal wall was demonstrated from the level of the cervical esophagus to the esophagogastric junction with fluid accumulation in both pleural cavities (Fig. 1). Esophagogastrography using water-soluble contrast medium injected through a nasogastric tube showed no evidence of esophageal perforation (Fig. 2). Esophagogastric endoscopy showed a normal appearance of the esophagogastric junctional mucosa. Based on these findings, she was diagnosed with acute phlegmonous esophagitis (APE).

The patient underwent drainage of the bilateral pleural effusions through chest tubes. The pleural fluid was serosanguinous without a purulent appearance. However, a bacterial culture of the pleural fluid that was taken from the right pleural cavity showed *Streptococcus milleri*.

She was immediately intubated and managed under mechanical ventilation in the ICU. Her vital status was unstable due to the persistent shock that was accompanied with DIC with an episode of cardiac arrest requiring cardiopulmonary resuscitation, as well as the intravenous administration of a large amount of catecholamines. Furthermore, broad-spectrum antibiotics, continuous hemodiafiltration, and polymyxin B-immobilized fiber hemoperfusion were needed to control the septic shock. The severe leukopenia persisted, despite the administration of granulocyte colony-stimulating factor (G-CSF), until the fifth day of the onset of the APE when the WBC count recovered to 6000 cells/μL.

**Fig. 3:**
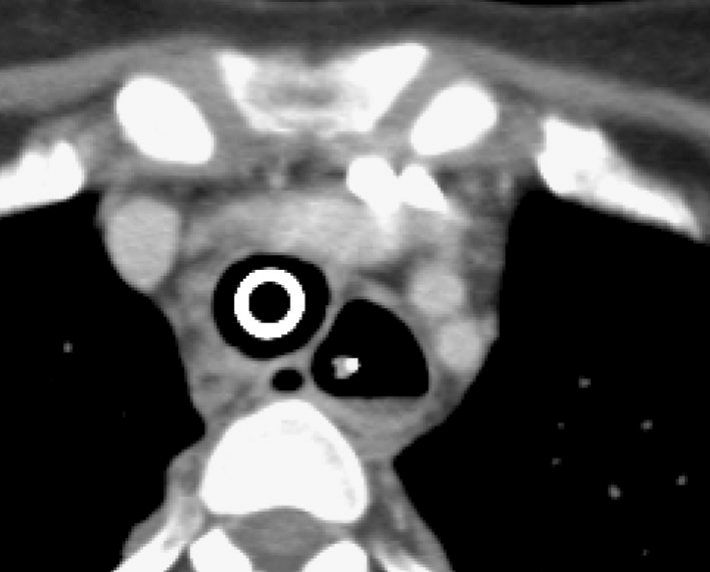
A follow-up computed tomography (CT) scan performed on the 15th day from the onset demonstrating the improvement in the diffuse esophageal wall thickening

**Fig. 4:**
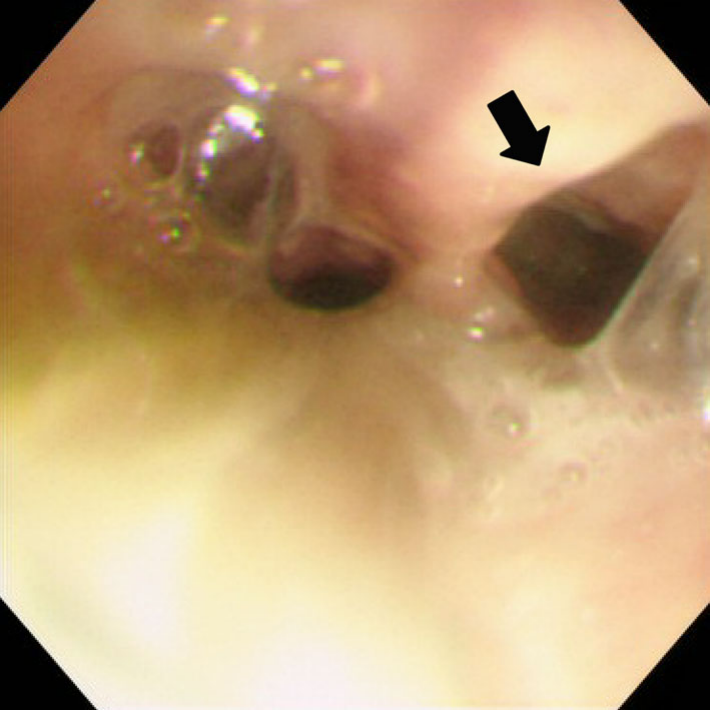
Upper gastrointestinal endoscopy performed on the 32nd day from the onset showing the esophageal mucosa peeling and pseudolumen formation (*an arrow*)

She recovered from the DIC and septic shock 2 weeks after the onset of the disease. A CT scan performed on the 15th day after the onset of the APE showed marked improvement of the edematous thickening of the esophageal wall (Fig. 3). On the 23rd day she underwent a jejunostomy to receive enteral nutrition. She was weaned from a mechanical ventilator on the 29th day. A transoral endoscopy performed on the 32nd day revealed mucosal peeling and pseudolumen formation of the esophagus at the level 15 cm distal from the esophageal inlet (Fig. 4).

**Fig. 5:**
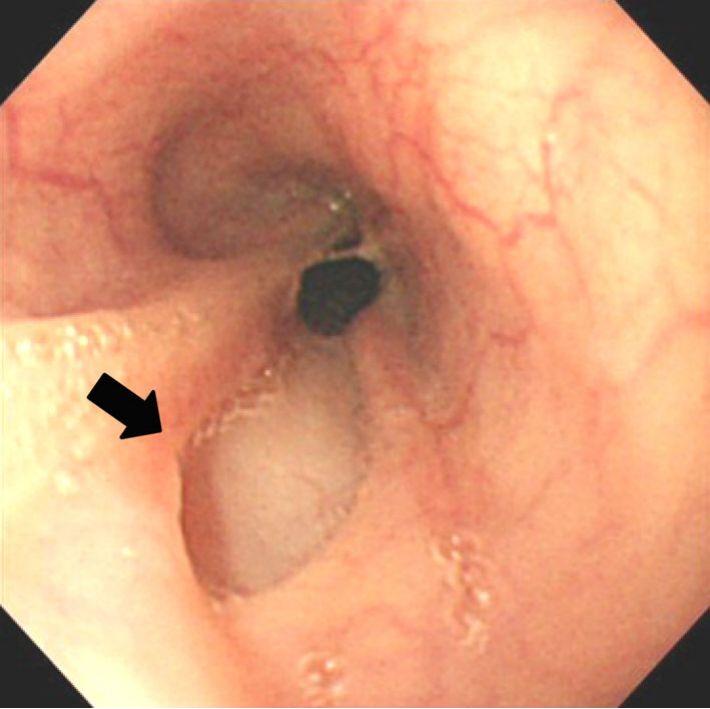
Upper gastrointestinal endoscopy performed on the 95th day from the onset showing an ulcer (*an arrow*) and circular stricture of the mid-esophagus with disappearance of the pseudolumen that had been observed previously

She underwent an amputation of the right lower leg on the 37th day because of severe ischemic changes caused by peripheral circulatory insufficiency. Hemodialysis was needed for 65 days for acute renal failure caused by the septic shock. Afterward, she gradually improved, and oral ingestion was started on the 74th day. Endoscopy performed on the 95th day showed an ulcer located just proximal to a circular stricture of the esophagus requiring pneumatic dilatation at the level 25 cm distal from the incisors with disappearance of pseudolumen of the esophagus (Fig. 5). A CT scan performed on the 135th day showed the disappearance of the inflammatory changes of the mediastinum and normalized appearance of the esophagus.

## Discussion

Acute phlegmonous esophagitis (APE) is an extremely rare condition. Our extensive survey of the literature using a PubMed Search with the keywords “phlegmonous esophagitis” found only 12 cases of APE with or without involvement of the neighboring stomach [2–13]. The clinical findings of these 12 cases are summarized in Table 1.

**Table 1:** Summary of the reported cases of phlegmonous esophagitis and the present case

No.	Age (years)/sex	Predisposing conditions	Symptoms	Period before treatment	Shock/DIC	Organ involved	Extent of disease	Causative pathogens	Primary treatment	Outcome	Ref. no.
1	62/M	Epiphrenic diverticulum, heavy drinker	Pain	3 weeks	−/−	E	Focal	*Enterobacter cloacae, Klebsiella pneumoniae*	Conservative	Alive	[2]
2	31/M	NP	Pain	2 days	−/−	S > E	Diffuse	*Bacteroides* spp., *Enterobacter cloacae*, *Klebsiella pneumoniae*	Conservative	Alive	[3]
3	42/M	NP	Pain, dyspnea	5 days	−/−	S > E	Diffuse	G(+) bac.	Surgery	Alive	[4]
4	49/M	Tonsillitis	Pain	3 days	+/+	S > E	Diffuse	Negative	Surgery	Alive	[5]
5	51/M	NP	Pain	5 days	−/−	E, S	Diffuse	Negative	Conservative	Alive	[6]
6	52/M	DM	Pain	2 days	−/−	E, S	Diffuse	G(+) bac.?	Conservative	Alive	[7]
7	41/M	Dental caries	Pain	10 days	−/−	E, S, D	Diffuse	*Peptostreptococcus micros*, *Fusobacterium* sp., α-*Streptococcus*, *Gemella morbillorum*	Conservative	Alive	[8]
8	63/F	DM	Pain	3 days	−/−	E	Diffuse	G(+) bac.	Conservative	Alive	[9]
9	73/F	NP	Pain	>7 days	−/−	E, S	Diffuse	α-*Streptococcus*?	Conservative	Alive	[10]
10	43/M	Heavy drinker	Pain	>14 days	−/+	E	Diffuse	*Klebsiella pneumoniae*	Conservative	Alive	[11]
11	62/M	DM	Pain	NA	−/−	E, S	Diffuse	α-*Streptococcus*	Conservative	Alive	[12]
12	48/M	Alcoholism, DM	Pain, dyspnea	3 days	−/−	E > S	Diffuse	*Klebsiella pneumoniae*	Surgery	Alive	[13]
Present case	47/F	CRT	Pain, dyspnea	10 h	+/+	E	Diffuse	*Streptococcus* *milleri*	Conservative	Alive	

*NP* nothing particular, *DM* diabetes mellitus, *CRT* chemoradiotherapy, *NA* not available, *E* esophagus, *S* stomach, *D* duodenum, *G(+) bac.* Gram-positive bacilli, *DIC* disseminated intravascular coagulation, *Ref. no.* reference number

The age of the patients ranged from 31 to 73 years with an average of 51.4 years. There were 10 males and two females. One-third of the 12 patients had uncontrolled diabetes mellitus as the predisposing condition. Only three patients had a responsible inflammatory focus in the oral cavity, pharynx, or esophagus. All of the patients had pain in the pharynx, retrosternal region, or epigastrium as an initial symptom. Dyspnea was also present in two patients. The time between the occurrence of the symptoms and the beginning of the treatment ranged from 2 days to 3 weeks, with a median of 5 days.

All but one of the patients had APE that diffusely expanded along the entire course of the esophagus. The stomach was involved in nine (75 %) of the 12 patients. Various pathogens have been reported as causative agents. However, the most common pathogen was *Klebsiella pneumonia*, which was found in one-third of the patients. *Streptococcus milleri* was considered the causative pathogen in the present case. The *Streptococcus milleri* group is part of the normal flora of the mouth, gastrointestinal tract, and genitourinary system. *Streptococcus milleri* has been reported to cause aspiration pneumonia in some cases [14]. Although *Streptococcus milleri* bacteria are weakly pathogenic, they may cause a lethal infection in cases in an immnocompromised host, as in the present case. Most of the patients presented in a severely ill condition, but only one and two patients had shock and DIC in the clinical course of APE, respectively. All 12 patients were successfully managed, and they survived after medical or surgical treatment. Therefore, the outcome after the treatment of APE is satisfactory, although intensive care is indispensable.

The most useful diagnostic modality was the CT scan that demonstrated the diffuse thickening of the esophageal wall and the edematous enlargement of the posterior mediastinum. Bilateral pleural effusions are commonly observed. All of these findings were confirmed in the present case (Fig. 1). A pseudolumen, ulcer, and circular stricture of the esophagus have been reported to develop subsequently in some cases [8, 10, 11]. These changes were also observed in our patient (Figs. 4, 5).

Compared with the 12 cases of APE reported previously, the clinical features of the present case were unique because of the fulminant progression after the postprandial vomiting, which was the onset event of the disease, to the established APE, which was associated with septic shock and DIC, in only 10 h. Furthermore, the severe leukopenia persisted during the first few days despite the administration of G-CSF, suggesting that the presence of myelosuppression due to the antecedent chemo- and radiotherapy was the predisposing condition of this disease.

The initial events of the APE were nausea and vomiting immediately after lunch, which were considered to be adverse effects of the chemoradiotherapy administered. Severe epigastralgia occurred 3 h later, and septic shock subsequently developed 10 h after the initial events. Therefore, it is plausible that the mucosal continuity might have been disrupted by an excessive elevation of the intraesophageal pressure during vomiting, which further facilitated the bacterial entrance into the esophageal wall. Possibly, the bacterial infection had progressed rapidly along the entire course of the esophagus due to the lack of defense mechanisms against infection in the host because of the myelosuppression caused by the antecedent chemoradiotherapy.

Intensive chemotherapy frequently evoking nausea and vomiting either alone or in combination with concurrent radiotherapy is one of the standard treatments for malignant tumors. Therefore, it is important for physicians to keep in mind that APE may occur as a life-threatening complication of chemoradiotherapy, although its incidence is extremely rare.
